# Strengthening HIV Programs in Sub‐Saharan Africa Through Implementation Research: A Mixed‐Methods Systematic Review

**DOI:** 10.1155/arat/7185732

**Published:** 2026-06-13

**Authors:** George Msema Bwire, Goodluck G. Nyondo, Belinda J. Njiro, Beatrice G. Aiko, Wigilya P. Mikomangwa, Martine A. Manguzu, Francis August, David T. Myemba, Rejea Magati, Pilly Chillo, Ferdinand Mugusi, Muhammad Bakari, Christopher Sudfeld, Raphael Z. Sangeda, Japhet Killewo

**Affiliations:** ^1^ Department of Pharmaceutical Microbiology, School of Pharmacy, Muhimbili University of Health and Allied Sciences, Dar es Salaam, Tanzania, muchs.ac.tz; ^2^ Department of Medicinal Chemistry, School of Public Health and Social Sciences, Muhimbili University of Health and Allied Sciences, Dar es Salaam, Tanzania, muchs.ac.tz; ^3^ Department of Epidemiology and Biostatistics, School of Public Health and Social Sciences, Muhimbili University of Health and Allied Sciences, Dar es Salaam, Tanzania, muchs.ac.tz; ^4^ MRC/Wits Rural Public Health and Health Transitions Research Unit, School of Public Health, University of the Witwatersrand, Johannesburg, South Africa, wits.ac.za; ^5^ Department of Pharmaceutics and Pharmacy Practice, School of Pharmacy, Muhimbili University of Health and Allied Sciences, Dar es Salaam, Tanzania, muchs.ac.tz; ^6^ Department of Clinical Pharmacy and Pharmacology, School of Pharmacy, Muhimbili University of Health and Allied Sciences, Dar es Salaam, Tanzania, muchs.ac.tz; ^7^ Department of Development Studies, School of Public Health and Social Sciences, Muhimbili University of Health and Allied Sciences, Dar es Salaam, Tanzania, muchs.ac.tz; ^8^ Department of Gender Studies, Mwalimu Nyerere Memorial Academy, Dar es Salaam, Tanzania; ^9^ Department of Internal Medicine, School of Clinical Medicine, Muhimbili University of Health and Allied Sciences, Dar es Salaam, Tanzania, muchs.ac.tz; ^10^ Department of Global Health and Population, Harvard T.H. Chan School of Public Health, Boston, Massachusetts, USA, harvard.edu

**Keywords:** evidence-based interventions, HIV/AIDS, implementation research, mixed methods, sub-Saharan Africa, systematic review

## Abstract

**Background:**

Resource‐limited settings face challenges in applying evidence to end the HIV/AIDS epidemic by 2030. Implementation research (IR) has emerged as a promising approach to translate evidence into practice. This review explores how IR has been utilized in sub‐Saharan Africa (SSA) to support efforts to end the HIV/AIDS epidemic.

**Methods:**

The review protocol was registered with PROSPERO (CRD42024515975). Articles were searched in PubMed, Embase, Scopus, and Web of Science, with a focus on HIV/AIDS evidence‐based intervention studies in SSA.

**Results:**

Out of the 2055 retrieved articles, 41 (2%) qualified for final analysis. The findings identify 11 key implementation strategies, including decentralized service delivery, task shifting, integrated HIV service delivery, capacity strengthening in diagnostics and treatment support, health system strengthening, promotion of pre‐exposure prophylaxis, prevention of vertical transmission, youth‐friendly services, community engagement, innovative technologies, and operational research. These approaches improved access to HIV testing, treatment, and prevention services, enhanced adherence and retention in care, and supported early diagnosis and continuity of care. However, implementation was influenced by cross‐cutting barriers such as limited resources and infrastructure, workforce constraints, inadequate training, stigma, weak coordination systems, and policy and funding limitations.

**Conclusion:**

IR provides a structured approach to identifying, adapting, and scaling effective HIV interventions within real‐world settings. The evidence highlights its role in supporting diverse strategies across health system levels while accounting for contextual barriers and facilitators. These findings highlight the importance of continued investment in IR and targeted capacity building to strengthen the effectiveness and sustainability of HIV/AIDS programs in SSA.

## 1. Background

Four decades since the onset of HIV/AIDS epidemic, an estimated 85.6 million individuals have been infected with HIV, resulting in approximately 40.4 million HIV‐related deaths [1]. As of 2022, an estimated 39 million people were living with HIV (PLHIV) globally, with approximately 1.3 million new infections. Furthermore, almost 76.4% of PLHIV globally were accessing antiretroviral therapy (ART), and 630,000 died from AIDS‐related causes in 2022 [2]. In this regard, HIV remains a public health concern, particularly in low‐ and middle‐income countries (LMICs) [3]. LMICs experience an unequal distribution of the global HIV burden, with sub‐Saharan Africa (SSA) being the most affected region, accounting for nearly two‐thirds of all PLHIV worldwide [2]. In these settings, the burden of HIV is often exacerbated by factors such as limited access to healthcare, economic disparities, stigma and discrimination, and insufficient resources for prevention, treatment, and care [4, 5].

To address the HIV epidemic, the international community has joined forces and set targets, including those outlined by UNAIDS and by Sustainable Development Goal (SDG) 3.3, which aims to end the AIDS epidemic by 2030 [6]. However, progress toward these targets has been uneven, with many LMICs, especially from SSA, struggling to achieve them due to various systemic challenges such as limited resources [2]. Conversely, a wealth of evidence has persistently emerged in the realm of HIV prevention, care, and treatment, driven by ongoing research efforts and technological advancements [7, 8]. This evidence encompasses a broad spectrum of interventions, ranging from biomedical strategies such as pre‐exposure prophylaxis (PrEP) to comprehensive care models integrating ART with mental health and social support services [9]. However, despite these advancements, challenges persist in translating evidence‐based interventions into scalable and sustainable programs that reach those most in need [10].

Implementation research (IR) has emerged as a critical approach in addressing the translation of evidence‐based interventions in HIV prevention, treatment, and care programs [10]. IR facilitates the translation of evidence into practice by identifying barriers and facilitators [11], adapting interventions to local contexts, testing effective implementation strategies [12], and informing policy and practice [13, 14]. Furthermore, IR ensures that interventions are not only effective but also feasible, acceptable, and sustainable in real‐world settings [12]. For instance, in prevention, studies evaluating the implementation of PrEP have informed strategies to overcome access barriers and ensure adherence, optimizing its impact on reducing HIV transmission [9, 15, 16]. Similarly, in treatment, research has improved the delivery of ART by identifying effective service delivery models and patient engagement strategies, leading to better ART initiation and retention rates [17]. Moreover, in care, integration of HIV services with other healthcare programs has been facilitated by IR, enhancing overall health outcomes for HIV‐positive individuals [18].

We conducted a systematic review to document how IR approaches have been utilized in the adoption of evidence‐based interventions aimed at ending the HIV/AIDS epidemic in SSA countries.

## 2. Method

### 2.1. Study Design

We formulated a systematic review protocol following the guidelines established by Preferred Reporting Items for Systematic Reviews and Meta‐Analyses Protocols (PRISMA‐P) [19] and registered it in PROSPERO (CRD42024515975). The review sought to explore how IR is being utilized to advance the goal of eliminating HIV/AIDS in LMICs, particularly in SSA. In this review, we searched for studies employing quantitative and qualitative methods to gain comprehensive insights into the implementation process and its impact [20].

### 2.2. Study Search

A search strategy was developed to retrieve articles indexed in PubMed, Scopus, Embase, and Web of Science from inception to February 21, 2024 [21]. Specifically for PubMed/MEDLINE, a search strategy was devised, incorporating keywords and Medical Subject Headings (MeSH) terms. The Boolean operator “AND” was employed to retrieve articles containing all specified keywords, while “OR” was utilized to gather results containing at least one of the keywords. The search query “(((“evidence‐based”[All Fields] AND (“intervention s”[All Fields] OR “interventions”[All Fields] OR “interventive”[All Fields] OR “methods”[MeSH Terms] OR “methods”[All Fields] OR “intervention”[All Fields] OR “interventional”[All Fields]) AND “HIV”[Title/Abstract] AND “implementation science”[Text Word]) OR “implementation research”[Text Word]) AND “HIV”[Title/Abstract]) OR (((“implementation science”[Text Word] OR “implementation research”[Text Word]) AND “HIV”[MeSH Terms]) OR (“effectiveness‐implementation”[All Fields] AND (“studies”[All Fields] OR “study”[All Fields] OR “study s”[All Fields] OR “studying”[All Fields] OR “study”[All Fields]) AND (“HIV”[MeSH Terms] OR “HIV”[All Fields])))” was used for PubMed then adapted to other databases. The search was limited to evidence indexed within the selected databases (PubMed, Scopus, Embase, and Web of Science), and no additional gray literature sources were systematically searched. No language restrictions were applied during the search.

### 2.3. Eligibility Criteria

In this review, we included studies targeting populations involved in HIV/AIDS prevention, treatment, or care in LMICs [22], with a particular focus on SSA countries. The review examined IR studies that focused on either interventions or strategies related to HIV/AIDS prevention, treatment, care, and support in LMICs, or studies that examined the process of implementing HIV/AIDS programs, policies, or interventions in LMICs. IR was operationalized as studies that explicitly evaluated the adoption, implementation, scale‐up, or sustainability of evidence‐based HIV interventions within real‐world settings, including the assessment of implementation outcomes such as feasibility, fidelity, acceptability, and effectiveness. Comparisons were made against conventional approaches or no intervention. The outcomes evaluated the effectiveness of the intervention on HIV/AIDS incidence and prevalence rates, uptake of HIV testing, treatment, and adherence to ART [8], among others.

We also investigated the implementation outcomes such as fidelity, feasibility, acceptability, and sustainability of interventions. Additionally, we included studies that reported on health system strengthening efforts related to HIV/AIDS care and support, and cost‐effectiveness and resource allocation for HIV/AIDS programs. Study designs (S) included studies that were IR, as described by Odeny et al. [22]. We excluded study designs, such as mathematical modeling, study protocols, case reports, correspondences, and letters to editors. Pilot studies were excluded to ensure the inclusion of studies with fully developed interventions and sufficient sample sizes to provide more robust and generalizable evidence on implementation outcomes; however, we acknowledge that this may have excluded early stage innovations and has been noted as a potential limitation. In our protocol, we made specific changes to include studies where the intervention (evidence) was both implemented and assessed within the same setting and population, or the study was a follow‐up of the implemented intervention.

### 2.4. Study Screening and Data Extraction

**FIGURE 1:**
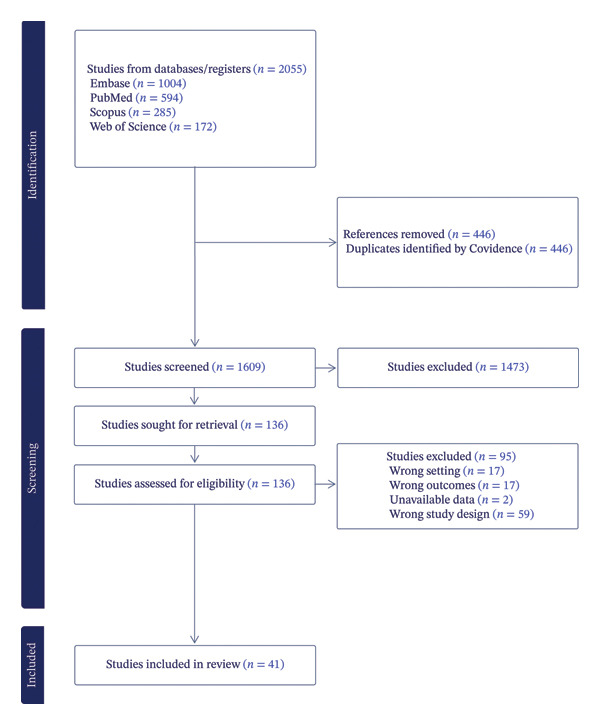
PRISMA flowchart showing the screening of 2055 articles leading to 41 remaining studies. Adapted from Covidence systematic review software (Veritas Health Innovation, Melbourne, Australia).

The Covidence systematic review software (Veritas Health Innovation, Melbourne, Australia) was used to manage the study selection process and identify any duplicate records. Two independent reviewers (GGN and BJN) assessed titles, abstracts, and full‐text articles to determine their eligibility for inclusion in the review (Figure 1). In the event of discrepancies, a third reviewer (GMB) was consulted to facilitate consensus. Relevant data, including publication year, country, study design, implemented interventions (evidence) or strategies, and outcome measures such as HIV testing rate, prevalence, incidence, retention in HIV care, and adherence to ART medications, were extracted from the eligible publications and entered in the Microsoft Excel Spreadsheet (Microsoft Corporation, Redmond, WA) as shown in Table 1. Our review focused on studies that reported the implementation of the interventions (evidence) or strategies in real‐world settings and assessed their outcomes.

**TABLE 1:** Characteristics of the reviewed studies and risk of bias assessment.

S/N	Study	Study design	Country	Population	Interventions or strategies	Effectiveness and/or implementation outcomes	Risk of bias
1.	Anne 2020 [54]	Longitudinal study	Kenya	Individuals who had recently tested HIV‐positive (*n* = 8,614)	‐Integrating an iris‐recognition biometric identification system into routine HIV care services ‐Innovative technologies and strategies)	Out of 6,078 return visits, the system accurately reidentified patients′ IDs 5,234 times (86%). The false match rate, where a new patient received another patient′s ID, was 0.5%, while the generalized false reject rate, where rescans led to new IDs, was 4.7%. Only nine individuals (0.1%) agreed to enroll but declined to undergo an iris scan, primarily due to concerns about privacy and confidentiality. Implementing an iris‐recognition system in routine health information systems is feasible and widely accepted as part of routine care in Kenya	Low
2.	Audet 2017 [30]	Mixed methods	Mozambique	Traditional healers, clinicians, and PLHIV	‐Engaging traditional healers as adherence partners for persons enrolled in HIV care and treatment ‐Task shifting ‐Community engagement and empowerment and health social determinants	Traditional healers, clinicians, and community members contributed ideas to customize the adherence support worker intervention, highlighting local attitudes such as HIV denialism, mistrust of the health system, and a preference for traditional treatments. Proposed changes included modifying training content and language, expanding community‐based activities for HIV acceptance and partner disclosure, and involving healers in promoting respectful clinical care. Focus groups confirmed the intervention′s social acceptability, leading to the successful enrollment of 180 newly diagnosed HIV‐infected patients, with a high acceptance rate of 94%.	Moderate
3.	Boehmer 2016 [42]	Cluster‐randomized trial	Nigeria	Patient and clinician (340 mothers, intervention *n* = 160; control *n* = 180 and 60 providers, intervention *n* = 36; control *n* = 24)	‐Strategies included task shifting to lower‐care workers, male engagement, point‐of‐care CD4+ cell counts, and integrated mother–infant care. ‐Task shifting	Intervention sites showed significantly higher patient satisfaction (median 4.61 vs. 3.84 in control; *p* < 0.001), while provider satisfaction was similar between intervention and control arms initially (median 3.60 vs. 3.50, *p* = 0.69). However, provider satisfaction decreased (median 3.47) when new roles were considered. Satisfaction levels weren′t linked to antiretroviral therapy uptake or mother‐infant retention at 6 and 12 weeks postpartum. Patients at intervention sites were more satisfied, but provider satisfaction dropped with new roles. Effective task shifting demands training and supportive oversight for comfort with assigned tasks.	Moderate
4.	Butler 2023 [66]	Mixed methods	South Africa	Adolescent girls and young women, aged 15–24 years	‐Strategies such as stakeholder engagement, routine data monitoring, facility assessment, training, and progress reporting were employed to increase access to oral PrEP for HIV prevention and sexual and reproductive health services. ‐Pre‐exposure prophylaxis (PrEP ‐Provision of youth‐friendly services. ‐Health system strengthening	Approximately 22,000 people were initiated on PrEP, of whom 67% were adolescent girls and young women. Stakeholder engagement ensured buy‐in, continuous staff training reinforced knowledge, and service models for adolescent girls and young women were contextually tailored.	Moderate
5.	Chikwari 2018 [60]	Randomized controlled trial	Zimbabwe	Children living with HIV and their caregivers (*n* = 172 in the intervention arm)	‐Community health worker (CHW)‐delivered support visits to children living with HIV and their caregivers ‐Task shifting ‐Community engagement and empowerment and health social determinants	The community‐based intervention proved to be a well‐received and viable strategy for lowering virological failure rates among children living with HIV.	Moderate
6.	Dovel1 2023 [39]	Cohort study	Malawi	Mothers living with HIV and infants (607 mother–infant pairs [MIPs]). The mean age of MIPs was 30 years and 7 weeks respectively	‐Integrated early childhood development (ECD) services and PMTCT intervention ‐Integrated HIV service delivery ‐Prevention of mother‐to‐child transmission of HIV (PMTCT)	Study found that 86% of mothers attended at least five out of eight ECD sessions within a year. Among intervention mothers, 88% remained in PMTCT, compared to 59% in other health facilities, and 96% of intervention infants were tested for HIV within six weeks, contrasting with 66% in nonintervention facilities. Cost analysis supported the financial viability of merging ECD and PMTCT programs in Malawi′s government health facilities. This integration proved feasible and acceptable and led to improved outcomes for both mothers and infants.	Moderate
7.	Eastment 2022 [27]	Qualitative nested study within the cluster‐randomized trial	Kenya	Family planning clinics (*n* = 24)	‐Systems Analysis and Improvement Approach (SAIA) ‐Integrated HIV service delivery	SAIA was successfully implemented across FP clinics of diverse sizes, capacities, and management support, demonstrating acceptability, appropriateness, and feasibility. The empowerment of clinic staff to propose and implement their own solutions contributed significantly to SAIA′s success.	Low
8.	Edwards 2016 [53]	Prospective quasiexperimental study	Kenya, Uganda, and South Africa	WHO Level 3, 4, and 5 healthcare institutions and their employed nurses	‐Establishing multistakeholder leadership hubs on evidence‐informed HIV care practices. ‐Health system strengthening	Leadership hubs, involving committed nurses and stakeholders trained in capacity building, can identify and act on strategies to enhance practice and policy. However, these hubs did not significantly improve the adoption of evidence‐informed HIV care in their districts. Integration within broader systems may be crucial for their success.	Low
9.	El Joueidi [46]	Mixed methods	Kenya and Rwanda	Key stakeholders (physicians, nurses, public health officers, etc.)	‐Implementation the mobile health platform “WelTel” ‐Innovative technologies and strategies	The thematic analysis identified strengths in timely diagnosis and response, cost‐effectiveness, and user‐friendliness. Areas for improvement highlighted were training, phone accessibility, stakeholder engagement, and literacy.	Low
10.	Fabian 2022 [57]	Mixed methods	Mozambique	Newly diagnosed HIV+ patients with comorbid mental health symptoms (*n* = 820).	‐Integrating a transdiagnostic psychological intervention (Common Elements Treatment Approach [CETA]) into routine HIV care ‐Integrated HIV service delivery	Approximately, 382 (46.6%) showed clinically significant mental health symptoms and attended 1484 (CETA) sessions. Incorporating CETA into routine HIV care has demonstrated feasibility within the given context	Moderate
11.	Georgeu 2012 [48]	Qualitative study	South Africa	Patients, health workers, health managers, and other key informants	‐Implementing nurse‐initiated and managed antiretroviral treatment (NIMART) ‐Task shifting	Scaling up NIMART in South Africa′s primary public health sector is feasible and well‐received. A successful implementation hinges on an incremental and well‐supported approach, nurse‐tailored clinical guidelines, and substantial health services reorganization to address practice shifts.	Low
12.	Gichane 2020 [63]	Mixed methods	South Africa	Women living with HIV and who use alcohol or other drugs, four cycles each cycle (*n* = 120)	‐The Women’s Health CoOp (WHC), a gender‐specific, risk‐reduction EBI designed for women dealing with alcohol and substance use. ‐Community engagement and empowerment and addressing the social determinants of health	All 100% of staff trained in the WHC delivered workshops, reporting its benefits and alignment with facility goals, but faced implementation challenges due to staff shortages, stigma, and limited referral options for supportive services; the cost was 1.40 USD per attendee, with interventionists maintaining high fidelity.	Moderate
13.	Haberer 2022 [58]	Pilot, mixed‐methods	Uganda	PLHIV on ART (*n* = 51) and social supporters (*n* = 48)	‐Electronic adherence monitors ‐Innovative technologies and strategies	A relatively low‐cost electronic ART adherence monitor, and associated interventions were successfully implemented for routine care in rural Uganda. Feasibility and acceptability were generally high.	Moderate
14.	Hassan 2019 [34]	Mixed methods	Tanzania	Health facilities and clients	‐Integrating methadone and ART (IMAT) program ‐Integrated HIV service delivery	The IMAT strategy implemented at an opioid treatment program clinic in Tanzania resulted in notable improvements in HIV services delivery. Key outcomes included a high reach, with 98% of PLHIV receiving services, and a significant increase in effectiveness, as demonstrated by the 90‐day ART initiation rate doubling from 41% to 87%. Additionally, the proportion of eligible clients on ART rose from 71% to 98%. The protocol also saw high adoption and implementation fidelity.	Low
15.	Herce 2020 [51]	Prospective cohort study (part of the larger multisite mixed‐methods study)	South Africa and Zambia	Incarcerated individuals aged 18 years or older (*n* = 975)	‐Implementation of universal test‐and‐treat (UTT) intervention in correctional facilities. ‐Integrated HIV service delivery	At the end of follow‐up, 86% of participants had started ART, with a median time of 0 days from enrollment to ART initiation. Among those remaining incarcerated at 6 months, 95% were retained in care, 78% had a documented viral load, and 97% achieved viral suppression. The mortality rate among participants who initiated ART was 1.9 per 100 person‐years. No statistically significant associations were found between baseline characteristics and time to ART initiation or composite poor outcome. Implementing UTT in correctional settings is feasible and can achieve same‐day ART uptake, retention in care, and viral suppression levels similar to those in community settings.	Low
16.	Iwelunmor 2022 [59]	Pilot, quasiexperimental cohort study	Nigeria	Adolescents and young adults aged 15–24 years (*n* = 388)	‐Crowdsourced youth‐led strategies to enhance HIV self‐testing (HIVST) ‐Provision of youth‐friendly services	Youth‐led HIVST interventions were found to be feasible and led to a notable increase in HIV/sexual transmitted infection test uptake.	Moderate
17.	Kumwenda 2023 [40]	Mixed methods	Malawi	Key stakeholders such as healthcare workers, male and female participants	‐Implementation of male involvement strategies to optimize the PMTCT program ‐Community engagement and empowerment and addressing the social determinants of health	The successful implementation of male involvement (MI) strategies was driven by factors like providers′ attitudes, coordinated service delivery, integrated training and services, and access to information, all of which positively impacted the outcomes for babies with HIV. However, challenges such as financial constraints, limited time, inadequate human resources, and the need for more male‐friendly spaces hindered the full implementation of MI strategies. To enhance MI strategies, a comprehensive systems approach is needed, addressing both health system and individual‐level factors for providers and consumers alike.	Low
18.	Long 2022 [36]	Cluster‐randomized trial	Kenya	Health facilities and healthcare providers	‐Systems analysis and improvement approach (SAIA) to increase HIV testing in family planning clinics. Clinics were randomly assigned to either the SAIA implementation strategy or standard care. During Stage 1, study staff managed all activities, while in Stage 2, we shifted SAIA implementation to department of health services (DOHS) staff and assessed HIV testing and counseling (HTC) rates in intervention and control clinics one year after the transition. ‐Integrated HIV service delivery	Despite facing challenges such as the COVID‐19 pandemic and a prolonged healthcare worker strike, which led to widespread healthcare disruptions and an incomplete implementation of the strategy, intervention clinics displayed sustained improvements in HTC even after SAIA was transitioned to DOHS leadership.	Moderate
19.	Mangale 2023 [29]	Mixed methods	Kenya	Healthcare providers	‐Provider‐driven adaptations made to support phone delivery of the adolescent transition package (ATP). ‐Innovative technologies and strategies	The study identified a total of 72 adaptations, with 32 being unique. These adaptations primarily focused on modifying context (53%), content (49%), and evaluation processes (13%). Context adaptations mainly involved changes to personnel, format, and setting, while content and evaluation adaptations often included simple additions, repetition, and tailoring of the phone delivery strategy. Interestingly, nine adaptations initially involved abandoning, then returning to phone delivery. Healthcare workers aimed to increase reach, improve fidelity, and ensure the intervention fit within their context. The majority of adaptations (96%) were perceived to enhance the feasibility of phone delivery compared to before the changes were implemented, with 83% of adaptations making phone delivery easier according to HCWs. These adaptations were mostly integrated into routine workflows (47%) or retested (47%). Adapting phone delivery proved to be a feasible and effective solution for addressing continuity of care challenges among young people living with HIV during the COVID‐19 pandemic. The majority of adaptations focused on contextual changes.	Moderate
20.	Medley 2023 [50]	Pre‐ and postimplementation study	Zambia	Women living with HIV (WLHIV) aged ≥16 years (629 WLHIV were interviewed preintervention and 684 postintervention)	‐Integrating family planning (FP) into existing HIV treatment services. ‐Integrated HIV service delivery	FP use increased significantly postintervention from 35% to 49%, with notable rises in injectables (15% to 25%) and implants (5% to 8%). Dual method use increased from 8% to 18%, and unmet FP needs decreased from 59% to 46%. Safer conception counseling uptake also improved from 27% to 39%. The intervention cost was estimated at $83,293. Integrating FP and HIV services can enhance women′s access to comprehensive sexual and reproductive health services.	Low
21.	Mithi 2023 [45]	Mixed methods	Malawi	PLHIV on ART (*n* = 100) and healthcare professionals	‐Implementing advanced HIV disease screening at a secondary referral hospital. ‐Diagnostic and treatment support and capacity strengthening. ‐Health system strengthening	The study identified significant contextual barriers to AHD screening, including knowledge gaps, poor communication systems, and inadequate supporting resources. Enhancing the uptake of AHD screening services would necessitate addressing these barriers through a comprehensive approach that develops strategies tailored to each barrier.	Moderate
22.	Mugo 2017 [43]	Cross‐sectional study	Kenya	Adult clients (≥18 years) seeking services indicative of HIV risk (*n* = 463).	‐Oral HIV self‐testing (HST) to community pharmacy clients. ‐Decentralized service delivery model	HIV testing uptake was significantly higher (84% vs. 11%) compared to other services. Only 4% of nontesters cited cost as a barrier. Almost all testers found the process easy. Poststudy, demand for HIV self‐testing kits remained, and service providers wanted to continue offering the service. HST in Kenyan pharmacies seems viable and potentially in high demand. A client‐initiated approach appears more feasible than a pharmacy‐led one. Pricing at around US$1 per test may not pose a barrier.	Low
23.	Nakambale 2023 [64]	Pilot, cross‐sectional study	Kenya	Pharmacy providers at five private pharmacies and remote clinician oversight.	‐Pharmacy‐delivered oral PrEP model for a fee of ∼3 USD using a prescribing checklist with remote clinician oversight. ‐Decentralized service delivery model. ‐Pre‐exposure prophylaxis (PrEP)	Pharmacy providers conducted screenings for 496 potential PrEP clients. Of these, 425 were deemed eligible for pharmacy‐delivered PrEP services, with 230 (54%) successfully initiated on PrEP. Additionally, among 197 clients eligible for PrEP continuation, 125 (63%) proceeded to refill their PrEP prescription. Implementation barriers included high costs for clients, client discomfort discussing sexual behaviors and HIV testing with providers, provider frustrations with time‐consuming PrEP delivery disrupting workflow, and provider hesitancy due to concerns about encouraging sexual promiscuity.	Low
24.	O’Malley 2014 [47]	Mixed methods	Namibia	Doctors, nurses, and patients	‐Nurse task shifting for ART services. ‐Task shifting	Findings indicated a high level of agreement (80%) between doctors and nurses on all dimensions of HIV care and 90% agreement on eight dimensions. To ensure the success of the national scale‐up of the task‐shifting model, potential challenges related to infrastructure, ongoing mentoring, and nursing scope of practice should be anticipated and proactively addressed	Moderate
25.	Omollo 2022 [31]	Pilot study, one‐arm randomized trial	Kenya	Pharmacies and clients	‐Pharmacy‐based oral HIV PrEP delivery model. ‐Decentralized service delivery model. ‐Pre‐exposure prophylaxis (PrEP)	A total of 287 clients‐initiated PrEP, with 159 (55%) returning for refills. During initiation, nearly all clients received counseling on PrEP adherence (99%) and potential side effects (97%), and all underwent provider‐assisted HIV self‐testing before receiving PrEP. Similar findings were observed during refill visits. Standardized client actors completed 15 pharmacy visits, where most were asked about HIV risk behaviors (80%) and all received counseling on PrEP safety and side effects.	Moderate
26.	Owiredu 2017 [28]	Mixed‐methods	Malawi, Nigeria, and Zimbabwe	Healthcare workers, researchers, and trainers	‐Strengthening health system capacity through implementation research: Insights from the multicountry PMTCT project (INSPIRE). ‐Operational research ‐Prevention of mother‐to‐child transmission of HIV (PMTCT)	INSPIRE created an opportunity for research led by Africans, collaborating closely with national Ministries of Health to identify and address priority research questions. Strong partnerships between research teams and local implementers allowed for quick responses to program challenges, leading to enhanced local program management and service delivery. This collaboration also resulted in improvements in data management, increased publications, and supported career development.	High
27.	Packel [56]	Case study, hybrid effectiveness implementation study (cluster RCT)	Tanzania	PLHIV on ART	‐Financial incentive intervention for HIV treatment adherence. Implementation research aided the implementation of three trials: one comparing cash and food assistance with standard care for ART adherence, another determining the optimal cash amount for improved viral suppression, and a third testing the optimized intervention′s effectiveness in various settings. ‐Community engagement and empowerment and addressing the social determinants of health	Implementation research enabled the bridging of the know‐do gap and facilitated the implementation of a multiphase optimization of financial incentive intervention for improving ART adherence and viral suppression among PLHIV. It also enabled to assess the utility, scalability, and sustainability of the intervention with the given context. Preliminary findings from the first trial showed that compared to standard care, cash and food assistance improved adherence among PLHIV on ART. Cash transfers were found to be superior, noninferior, less expensive, and easier to implement than food assistance	Moderate
28.	Roberts 2023 [52]	Mixed methods	South Africa, Uganda, and Zimbabwe	Adolescent girls and young women (247 HIV‐negative aged 16–21years)	‐Adherence to the ring and oral PrEP. Adherence support strategies included monthly counseling sessions with drug‐level feedback (DLF) plus optional daily short message service (SMS) reminders, weekly phone or SMS check‐ins, peer support clubs, “peer buddies” and additional counseling. ‐Pre‐exposure prophylaxis (PrEP) ‐Provision of youth‐friendly services ‐Diagnostic and treatment support and capacity strengthening	Effectiveness was underpinned by three key factors: robust interpersonal relationships with counselors, readily available ongoing support and resources, and the establishment of trust in both counselors and study products through counselor relationships, peer‐to‐peer exchange, and DLF. To support effective PrEP use, implementation programs can offer youth‐friendly and developmentally appropriate counselor‐ and peer‐based support options	Low
29.	Roche 2021 [37]	Mixed methods	Kenya	PrEP clients and providers	‐A one‐stop shop (OSS) model for improved efficiency of PrEP delivery in public clinics ‐Pre‐exposure prophylaxis (PrEP)	An OSS model substantially reduced client wait times and enhanced care acceptability while maintaining initiation and continuation rates, underscoring the potential to enhance PrEP delivery efficiency and client‐centeredness.	Moderate
30.	Saldarriaga 2024 [62]	Mixed methods	Kenya	Adolescents and young adults aged 15–24 living with HIV (13 HIV clinics)	‐Implementation cost for adolescent transition package (ATP). ATP is an EBI to improve the readiness of young people (15 to 24 years) with HIV to transition into HIV adult care. ‐Provision of youth‐friendly services	The average cost per adolescent HIV care visit was 29.8 USD (95% CI 27.5, 33.4) in the standard‐of‐care arm and 32.9 USD (95% CI 30.5, 36.8) in the ATP intervention arm, resulting in an incremental cost of 3.1 USD (95% CI 3.0, 3.4) for the ATP intervention. The ATP can be feasibly implemented in HIV care clinics with a slight increase in overall clinic visit costs.	Moderate
31.	Sharma 2023 [41]	Single‐arm, hybrid type 2 implementation‐effectiveness study	Kenya	Male partners of female individuals (aged ≥ 18 years or emancipated minor) who tested positive for HIV (*n* = 19,100 female individuals tested positive for HIV)	‐Integrating HIV assisted partner services (APSs) into health facilities ‐Integrated HIV service delivery ‐Community engagement and empowerment and addressing the social determinants of health	The study revealed that out of 5137 male partners identified by female index clients (median of 3 partners per index, IQR 2–4), 86% were reached through exposure notification and HIV testing services (HTS). Among tested partners, 12% were newly diagnosed with HIV, and 29% had a previous HIV diagnosis. After 12 months, 88% of female index clients and 89% of male partners with HIV were taking ART. Adverse events were low, with 2% of female index clients and < 1% of male partners reporting intimate partner violence, and 3% of female index clients and < 1% of male partners reporting relationship dissolution. The findings of the real‐world APS scale‐up project demonstrated that APS had been a dependable, widely accepted, and effective approach for identifying HIV‐positive males and ensuring their sustained engagement in care	Low
32.	Sisimayi 2023 [38]	Mixed methods	Zimbabwe	Adolescent girls and young women (AGYW)	‐“V”: a multilevel PrEP intervention designed with and for adolescent girls and young women. “V” is a branding and service strategy that encourages AGYW to empower themselves by shifting the focus from avoiding HIV to embracing oral PrEP uptake and continuation ‐Pre‐exposure prophylaxis (PrEP) ‐Provision of youth‐friendly services	Providers and young women found “V” to be appealing because of its attractive branding and informative messaging, which created a comfortable space for discussing PrEP and filled a communication gap. Integration of “V” into routine services and outreach for adolescent girls and young women (AGYW) was feasible and complemented existing services. The cost analysis highlighted the importance of essential “V” branded materials such as the FAQ insert, pill case, makeup bag, and reminder sticker, which amounted to $7.61 per AGYW initiated on PrEP	High
33.	Spooner 2019 [55]	Hybrid type‐2 effectiveness implementation study (observational study)	South Africa	HIV‐exposed infants for birth PCR testing in hospital (*n* = 323) and follow‐up at a primary healthcare clinic (*n* = 117)	‐Point‐of‐care (POC) HIV testing of infants compared to standard‐of‐care (SOC) central‐laboratory testing. ‐Diagnostic and treatment support and capacity strengthening	The POC error rate was 9.6%, with all cases providing a result upon repetition. A large majority of mothers (92%) favored POC testing, with only 7% expressing no preference. Conversely, no staff members preferred SOC testing, with 79% preferring POC and 21% having no preference. POC HIV testing for early infant diagnosis is accurate, feasible, and beneficial, allowing early ART initiation for positive infants at birth facilities	Moderate
34.	Thomas 2022 [26]	Mixed methods	Uganda	Health facilities	‐Integrated model of oral PrEP and ART delivery for HIV sero‐discordant couples ‐Integrated HIV service delivery ‐Pre‐exposure prophylaxis (PrEP)	Important implementation facilitators for PrEP included educating facility staff about it, setting up feedback mechanisms, and empowering staff to tackle challenges. However, barriers like ineffective recruitment of sero‐discordant couples and stockouts of testing supplies hindered implementation	Low
35.	Tollefson 2023 [44]	Mixed methods	South Africa	Healthcare professionals	‐Leveraging lay health workers (LHWs) for HIV response: Youth Health Africa (YHA) is an innovative approach in South Africa, placing young adults in one‐year nonclinical internships at health facilities to support HIV programs as testers, data clerks, and more ‐Task shifting	Nearly all 33 frontline healthcare workers interviewed found the YHA program highly acceptable and appropriate. Their positive perceptions, including mutual benefit, ease of integration, and enhanced facility success, contributed to its strong acceptability and appropriateness. However, concerns about training interns and limited program communication tempered this.	Moderate
36.	Van Rie 2014 [33]	Cross‐sectional study	South Africa	Primary care facilities	‐Systematic tuberculosis (TB) symptom screening and HIV counseling and testing (HCT) for all adult clients at a primary care clinic ‐Integrated HIV service delivery	During the first 6 months, the clinic saw 26,515 visits from 12,078 adults, with HIV awareness increasing from 43.7% to 90%, 1042 new HIV diagnoses, and a prevalence of 22.9% among newly tested individuals and 58.9% overall. Systematic HIV testing remained consistently high, but TB symptom screening declined after the initial period, dropping to 70% coverage and 15% assessment by sputum. Routine, systematic HCT and HIV‐stratified TB symptom screening have demonstrated feasibility and effectiveness at the primary care level.	Low
37.	Wagner 2021 [67]	Hybrid, cluster RCT	Uganda	HIV clients considering childbearing with an HIV‐negative partner (*n* = 389)	‐Safer conception counseling (SCC) to promote the use of safer conception methods (SCM). Using either a high (SCC1)‐ or low‐intensity (SCC2) approach (differentiated by amount of training and supervision), or existing family planning services (standard of care). ‐Community engagement and empowerment and addressing the social determinants of health	The combined intervention groups showed significantly higher usage of appropriate reproductive methods compared to usual care (20.8% vs. 6.9%), with SCC1 reporting a higher rate than SCC2 (27.1% vs. 14.6%). Among those trying to conceive, the intervention arms demonstrated greater accurate use of SCM compared to usual care (24.1% vs. 0%), and SCC1 outperformed SCC2 (34.6% vs. 11.5%). However, there were no differences in modern contraception use among those not trying to conceive. SCC1 incurred a cost of $631 per person to achieve accurate use of SCM, whereas SCC2 had a higher cost of $1014 per person for the same outcome. Intensive training and frequent supervision boost complex SCM adoption, proving more cost‐effective than low‐intensity methods.	Moderate
38.	Willard‑Grace 2024 [35]	Qualitative	Tanzania	Adolescent girls and young women (AGYW)	‐Implementing the Malkia Klabu program to improve access to HIV self‑testing and contraception. The Malkia Klabu initiative redesigned the role of drug shopkeepers, empowering them to offer information and resources instead of acting solely as gatekeepers for family planning and reproductive health (FPRH) services. ‐Task shifting ‐Community engagement and empowerment and addressing the social determinants of health)	The Malkia Klabu program transformed drug shopkeepers into information providers for FPRH instead of gatekeepers. Its success factors included adaptability, leveraging AGYW social networks, respecting privacy, boosting income and community status for shopkeepers, and introducing components like HIV self‐testing and loyalty programs that enhanced product provision and information sharing. While some shopkeepers initially had reservations about certain contraceptive methods, most integrated them into the program	Low
39.	Zakumumpa 2016 [49]	Mixed methods	Uganda	A nationally representative sample of health facilities (*n* = 195) and in‐depth interviews (*n* = 36) with ART clinic managers and staff	‐Strategies identified included providing incentives, spacing appointments, offering training workshops, adopting nonphysician staffing models, and enhancing leadership styles to boost health worker commitment in ART programs. ‐Diagnostic and treatment support and capacity strengthening ‐Health system strengthening	Implementing facility‐level strategies for human resources for health constraints is feasible and aids in increasing country ownership of HIV programs in resource‐limited settings. These strategies, identified by ART program planners and managers, can enhance the long‐term sustainability of ART programs among providers in such settings.	Moderate
40.	Zakumumpa 2017 [65]	Mixed methods	Uganda	In the first phase, nationally representative health facilities (*n* = 195). In the second phase, interviews with clinic managers (*n* = 9)	‐Reducing the frequency of clinic appointments and pharmacy‐only refill programs, implementing home‐based care programs, task shifting to nonphysician cadre ‐Task shifting ‐Decentralized service delivery model	Reducing clinic visits and implementing pharmacy‐only refills were identified as key strategies for decongesting ART clinics. Home‐based care programs were introduced to reduce provider ART delivery costs. Task shifting to nonphysician cadre was reported in 181 (93%) of the health facilities. Visits to the ART clinic were rationalized in favor of the subpopulation deemed to have more clinical need. The modifications were made to improve the fit with their resource‐constrained settings, thereby promoting long‐term sustainability.	Moderate
41.	Zakumumpa 2021 [32]	Case study, qualitative	Uganda	Ministries of Finance, Health, and Public Service (*n*=14), representatives of PEPFAR implementing organizations (*n*=16), district health teams (*n*=15) and facility managers (*n*=22) and 87 health workers absorbed on government payrolls.	‐Transitioning health workers from President′s Emergency Plan for AIDS Relief (PEPFAR) contracts to the government payroll ‐Health system strengthening	In “high absorber” districts at the subnational level, successful transition was facilitated by having transition champions, prioritizing healthcare workers in district wage bill commitments, host facilities providing bridge financing during salary delays, and receiving donor technical support for wage bill analysis—features notably lacking in ′low absorber’s districts. On a national scale, engaging multiple sectors (including the influential Ministry of Finance), developing a collaborative transition roadmap, and aligning with government salary scales and recruitment processes were identified as key facilitators. These findings provide valuable insights into effective donor transition strategies and offer practical approaches to enhance public spending for expanding the health workforce in low‐income settings	Low

Abbreviation: ART: Antiretroviral therapy; EBI: evidence‐based intervention; PCR: polymerase chain reaction; PLHIV: people living with HIV; PMTCT: prevention of mother‐to‐child transmission of HIV; PrEP: pre‐exposure prophylaxis; RCT: randomized controlled trial; WHO: World Health Organization.

## 3. Methodological Quality

Two independent reviewers (GGN and BJN) assessed the risk of bias (methodological quality) of the included studies. In the event of discrepancies, a third reviewer (GMB) was consulted. We used the Mixed Methods Appraisal Tool (MMAT), which has demonstrated utility in systemic reviews that involve various study designs to assess the risk of bias of the included studies [23]. This tool provided a structured approach to evaluating the methodological quality of qualitative studies, quantitative randomized controlled trials, quantitative nonrandomized, quantitative descriptive, and mixed‐methods studies. The tool includes two screening questions and outlines four criteria for the evaluation of the methodological quality of the specific design, including research questions, methods, outcome assessment, and integration of findings from quantitative and qualitative components for mixed‐method studies. For each study design, a total of five items were evaluated, with a maximum of five scores; each item received a score of either 1 for “Yes” or 0 for “No” or “Can’t Tell.” Total scores of 1, 2‐3, and 4‐5 were categorized as high, moderate, and low risk of bias, respectively [24].

### 3.1. Data Analysis and Narrative Synthesis

The outcomes of the included studies were summarized using proportions and other outcome measures as reported in the original articles, where appropriate. For studies with comparable quantitative data, descriptive statistics (e.g., proportions and percentages) were used to summarize key implementation and effectiveness outcomes. However, due to substantial heterogeneity in study designs, populations, interventions, and outcome reporting, a formal meta‐analysis was not feasible.

A convergent integrated mixed‐methods approach was employed, consistent with established guidance for mixed‐methods systematic reviews (e.g., JBI methodology) [25]. Quantitative findings were transformed into narrative summaries and integrated with qualitative data through thematic synthesis. This process involved coding and grouping findings across studies into key themes related to implementation strategies, processes, barriers, facilitators, and outcomes. The integrated synthesis enabled comparison and triangulation of evidence from both qualitative and quantitative studies to provide a comprehensive understanding of how implementation strategies influence the adoption, adaptation, and scale‐up of HIV/AIDS interventions across diverse contexts. This approach facilitated the identification of key lessons, best practices, and policy‐relevant insights for strengthening HIV programs in resource‐constrained settings.

## 4. Results

### 4.1. Characteristics of the Included Studies

A total of 2055 articles were initially retrieved from the searched databases. After applying the eligibility criteria, 42 studies [26–61, 63–67] were found to meet the inclusion criteria for the review. In this review, the majority of the studies (24 studies) were implemented in Kenya [27, 29, 31, 35–37, 43, 46, 53–55], Uganda [26, 32, 41, 49, 58, 65, 67], and South Africa [33, 44, 48, 52, 63, 66]. Some of the studies explored strategies in HIV prevention and care. For example, Mugo et al. investigated oral HIV self‐testing in community pharmacies [43]. Additionally, the implementation of a pharmacy‐based model for delivering oral HIV PrEP was described by Omollo et al. 2017 [31]. In the realm of HIV treatment, several studies explored diverse strategies to improve care and outcomes; this includes task shifting [42] scaling up nurse‐initiated and managed ART [48], and integration of ART with other services such as PrEP [26]. In the domain of HIV care, various studies examined innovative approaches to enhance services and support for individuals affected by HIV. For instance, Edwards et al. (2016) [53] focused on establishing leadership hubs for evidence‐informed HIV care, aiming to improve decision‐making and resource allocation within healthcare settings. The included studies comprised 19 mixed‐methods studies, 5 quantitative randomized controlled trials, 10 quantitative nonrandomized studies, 2 quantitative descriptive studies, and 5 qualitative studies (Table 1). The details on the risk of bias can be found in Supporting file 1.

### 4.2. Implementation Strategies

Eleven main implementation strategies were identified throughout this review.

#### 4.2.1. Decentralized Service Delivery (DSD) Model

Four studies employed this technique, targeting interventions that aim to minimize the burden on clients and to decongest higher‐level health facilities. The implementation of differentiated service delivery models such as pharmacy‐based HIV care and treatment services, including oral HIV self‐testing [43, 65] and PrEP, were found to be feasible for decentralization in resource‐limited settings. However, Mugo et al. (2017) [43] cited cost to the client as a potential barrier. Other implementation barriers included discomfort in discussing sexual health, provider frustration over PrEP’s time demands, including providing counseling on safety and side effects [31], and hesitancy due to worries about promoting risky behaviors [64].

#### 4.2.2. Task Shifting

Task shifting in HIV services strategy was implemented in eight studies and used to optimize healthcare resources and enhance access to quality care. Tasks shifting to lower cadre [42] and implementing nurse‐initiated HIV services [44, 47, 48] were feasible approaches to scale up HIV treatment and care services, resulting in improved health outcomes and a more sustainable healthcare system. Additionally, delegating specific tasks from highly specialized professionals to less specialized healthcare workers, such as community health workers and nurses, facilitated the reach of underserved populations, reduced costs, alleviated the workload of specialized professionals, and reduced congestion at the health facilities [30, 35, 44, 59, 60, 65]. Although task shifting improved access and efficiency in HIV services, several barriers were reported, including limited training and supervision for lower‐cadre workers, resistance from specialized professionals, restrictive policies, and increased workload for nurses and community health workers. Resource constraints, weak coordination systems, and concerns about patient trust in less specialized providers also affected effective implementation.

#### 4.2.3. Integrated HIV Service Delivery

This approach was reported in 10 studies and involved combining prevention, testing, treatment, care, and support services to address the complex needs of individuals living with or at risk of HIV/AIDS. For instance, integrating HIV testing and counseling with primary healthcare services such as family planning [27, 36, 50], PrEP [26], maternal and child health [39, 41], tuberculosis screening [33], and mental health support [34, 57] improved access, increased efficiency, enhanced continuity of care, and reduced stigma. Furthermore, IR has accelerated the adoption of the “universal test‐and‐treat” strategy in HIV prevention and management efforts through integration by providing evidence‐based frameworks, strategies, and tools tailored to address key barriers and facilitators in real‐world settings [51]. Common barriers to integrated HIV service delivery include limited resources and infrastructure, staff shortages, increased workload, and inadequate training. Fragmented health systems and funding, weak coordination and data systems, and unclear policies further hinder implementation, while stigma and low patient awareness reduce service uptake.

#### 4.2.4. Capacity Strengthening in Diagnostic and Treatment Support

Four studies reported that implementing interventions to increase health facility capacity for timely and effective treatment, specifically through point‐of‐care testing and adherence support, has been associated with measurable improvements in healthcare outcomes within the context of HIV/AIDS management [28, 45, 49, 62]. Point‐of‐care testing [55] has demonstrated a reduction in turnaround times for diagnostic results, facilitating timely treatment initiation and positively impacting patient satisfaction metrics. Simultaneously, adherence support programs have yielded tangible benefits, including enhanced medication adherence among individuals living with HIV/AIDS, resulting in observable reductions in viral load levels and improved treatment adherence rates [51, 52]. Some barriers to capacity strengthening in diagnostic and treatment support include limited availability of equipment and supplies for point‐of‐care testing, inadequate training of healthcare workers, and challenges in maintaining quality assurance. Weak supply chains, infrastructure constraints (such as unreliable electricity), and increased staff workload can further hinder implementation, while poor patient follow‐up systems and limited resources for adherence support programs may reduce overall effectiveness.

#### 4.2.5. Health System Strengthening

Five studies reported on the implementation barriers and facilitators to inform the development of different strategies for relevant interventions considering contextual factors at health system levels. Implementation frameworks were used to identify barriers to the expansion of advanced HIV disease screening at district hospitals, such as poor work coordination, limited resources, and knowledge gaps among providers [45]. IR has also been used to document and address barriers and constraints at different levels of health systems. For example, multistakeholder leadership training and workshops, incentives, and nonphysician staffing models implemented for health providers led to improvement in the adoption of evidence‐based HIV care practice and commitment to ART programs [49, 53]. Addressing implementation barriers in Uganda similarly proved successful in fostering effective donor transition and expanding the health workforce [32]. Furthermore, continuous staff training, facility assessments, and monitoring were successful implementation strategies for increasing oral PrEP access and utilization [66].

#### 4.2.6. PrEP

Eight studies reported interventions promoting access to and awareness of PrEP for high‐risk individuals as one of the key strategies in HIV prevention. The studies have shown that successful implementation of PrEP programs can lead to positive implementation outcomes, such as increased uptake rates among the target population, improved adherence to PrEP regimens, and enhanced awareness of PrEP as a preventive option [28, 38, 52]. In the implementation of PrEP programs, barriers such as cost constraints, stigma surrounding HIV/AIDS and PrEP, limited healthcare provider knowledge, and access issues have been identified [31, 64]. Conversely, facilitators include targeted awareness campaigns, affordability programs, healthcare provider training, and integrated services [26, 37], peer support and counseling initiatives, and supportive policies [66].

#### 4.2.7. Prevention of Vertical Transmission (PVT) of HIV

Optimization of service delivery through IR has led to improved accessibility, continuity of care, and quality of services provided to pregnant women living with HIV and their infants [28, 39]. A study by Boehmer et al. (2016) demonstrated how IR has informed the adaptation of PVT interventions to local contexts, leading to enhanced program effectiveness and scalability of best practices [41]. Conversely, barriers such as limited access to healthcare facilities, particularly in rural or underserved areas, can hinder pregnant women from accessing PVT services. Additionally, fear of stigma and discrimination associated with HIV/AIDS may discourage women from seeking HIV testing and treatment during pregnancy.

#### 4.2.8. Provision of Youth‐Friendly Services

Five studies reported the development and adoption of youth‐friendly HIV services. Such services are tailored to the specific needs of young people, leading to improved engagement in care, increased HIV testing rates, facilitated early linkage to treatment for those who tested positive [35, 38, 59, 66], and successful transition of adolescents into adult HIV care [62]. For example, adherence support strategies encompassed monthly counseling sessions, alongside daily short message service (SMS) reminders, weekly phone or SMS check‐ins, and participation in peer support clubs improved adherence to the ring and oral PrEP [52]. Implementation barriers include limited funding and resources to sustain youth‐specific programs, inadequate training of healthcare providers in adolescent‐friendly care, and concerns around privacy and confidentiality. Stigma, cultural norms, and low trust or awareness among youth can also limit uptake, while logistical challenges such as clinic hours, accessibility, and retention in care further hinder effective implementation.

#### 4.2.9. Addressing Social Determinants of Health Through Community Engagement and Empowerment

Community engagement and empowerment approaches were reported in eight studies. These interventions create a supportive environment that strengthens HIV prevention, care, and treatment efforts and contributes to improved health outcomes at both individual and population levels [30, 60]. This involvement facilitated targeted outreach efforts, reduced stigma and discrimination, enhanced HIV education and risk‐reduction strategies, encouraged HIV testing and linkage to care [35, 67], supported adherence to treatment regimens, and promoted community‐driven advocacy for policies and programs that address the needs of people living with HIV/AIDS. IR identified partner support and integrated services as the facilitators [41]. Additionally, IR played a crucial role in addressing social factors such as poverty [40, 56], stigma [63], discrimination, lack of education, and limited access to healthcare on HIV outcomes [35], which were important for achieving equitable and effective HIV care and prevention. Limited funding and sustainability of community‐based programs, inadequate training and support for community health workers, and weak coordination between communities and formal health systems are key barriers. Stigma, discrimination, and cultural norms may also hinder participation, while low community trust, poverty, and limited education can reduce engagement and uptake of services.

#### 4.2.10. Innovative Technologies and Strategies

Four studies employed innovative technologies and strategies as implementation strategies at different levels of HIV care. Embracing innovations such as mobile health (mHealth) interventions have been shown to enhance service delivery and reach marginalized populations, fostering continuity of care and adolescent care transitions [50]. Furthermore, innovative technologies and strategies have significantly contributed to HIV prevention and care by enhancing accessibility, accuracy, and efficiency in HIV testing, treatment, and support services [46]. A study conducted in Kenya reported that integrating an iris‐recognition biometric identification system into routine HIV care services was acceptable and feasible [54]. However, concerns about privacy and confidentiality emerged as barriers. Low‐cost electronic ART adherence monitors and associated interventions were successfully implemented in low‐resource settings [58].

#### 4.2.11. Operational Research

Two studies highlighted the vital role of investing in operational research for data monitoring and evaluation in HIV programs to strengthen healthcare systems, improving outcomes, and informing evidence‐based decision‐making [27]. This approach enables healthcare providers and policymakers to identify best practices, tailor interventions to specific needs, address implementation challenges, and scale up successful strategies, ultimately leading to more efficient and impactful HIV prevention and care programs. Additionally, strengthening health system capacity through IR resulted in improvements in data management, increased publications, and supported career development [28]. Barriers included limited funding for research activities, inadequate technical capacity for data collection and analysis, and weak data management systems. Poor data quality, limited use of data for decision‐making, and a shortage of trained personnel further hinder effectiveness, while institutional and policy constraints may restrict the integration of research findings into practice.

## 5. Discussion

Despite the emergence of numerous effective strategies to combat HIV/AIDS on a global scale [68–71], the adoption of evidence‐based practices remains a significant challenge, particularly in SSA [72]. This challenge is exacerbated by limitations in healthcare infrastructure and resources [73], which limit the consistent delivery of high‐quality HIV prevention, testing, and treatment services in the region [74, 75]. To address this challenge, IR efforts can focus on innovative strategies such as DSD models. The scientific findings from this review demonstrate both the acceptance and feasibility of implementing this approach, highlighting its sustainability even in low‐resource settings. Decentralized models offer advantages such as increased accessibility and reduced travel burdens, particularly benefiting underserved populations. Some of the challenges identified encompass linkage to care. Additionally, obstacles arise from stigma and inadequate funding for providers to effectively establish community drug pick‐up points. These findings are consistent with existing literature demonstrating that decentralization improves service uptake but requires strong referral and follow‐up systems to ensure continuity of care. Importantly, the persistence of linkage gaps suggests that decentralization alone is insufficient and must be complemented by community‐based support and health system strengthening to achieve optimal outcomes. For HIV programs, this implies that scaling up decentralized models should be accompanied by investments in community health systems and financing mechanisms to sustain service delivery.

Task shifting, a strategy in healthcare management, redistributes specific responsibilities from higher‐skilled healthcare professionals to lower‐skilled or nonspecialized personnel, thus enhancing the efficiency and accessibility of healthcare services, particularly in resource‐constrained environments. Findings from this review have shown that the implementation of task shifting, particularly testing and treatment to lower‐cadre healthcare workers, and implementing nurse‐initiated HIV services are feasible, cost‐effective, and sustainable. This approach facilitates reaching underserved populations, reduces costs, and alleviates the workload of specialized professionals. Similar to its success in HIV care as described in this review, task shifting has demonstrated effectiveness in the management of hepatitis C virus (HCV) infection. The delegation of care and treatment tasks to nonspecialists has shown comparable cure rates to those achieved by specialists, across various populations and healthcare environments, indicating its effectiveness and adaptability [76]. These findings reinforce broader evidence that task shifting is a critical strategy for addressing human resource shortages in LMICs. However, its effectiveness is contingent on adequate training, supervision, and regulatory support. Without these enabling conditions, task shifting may compromise quality of care. Therefore, HIV programs should prioritize structured training, supportive supervision, and clear policy frameworks to maximize the benefits of task shifting while maintaining care standards.

In this review, integrated HIV service delivery was implemented, and strategies to strengthen health systems were identified, resulting in promising implementation outcomes and underscoring the necessity for scaling up these approaches. Integrated HIV service delivery, combining prevention, testing, treatment, care, and support services, addresses complex needs effectively [77]. Integrating HIV services with primary healthcare services including chronic disease, PrEP services, family planning, maternal and child health, tuberculosis screening, and mental health support improved access, efficiency, continuity of care, and reduced stigma. Additionally, the implementation of point‐of‐care testing including infants and adherence assistance not only reduced turnaround times but also facilitated prompt treatment initiation, further enhancing the test‐and‐treat policy as a preventive measure. On the other hand, IR facilitated the contextual adoption of PrEP programs, leading to increased uptake rates, improved adherence, and enhanced awareness of PrEP as a preventive option. Barriers such as cost constraints and stigma need addressing through targeted awareness campaigns, affordability programs, healthcare provider training, integrated services, and supportive policies. These findings align with prior research emphasizing that service integration enhances health system efficiency and patient‐centered care. However, the variability in implementation outcomes across settings highlights the importance of contextual adaptation. This suggests that “one‐size‐fits‐all” models are unlikely to succeed, and HIV programs must tailor integrated service delivery approaches to local health system capacities, population needs, and resource availability.

Optimization of service delivery through IR has improved accessibility, continuity, and quality of PMTCT services. Facilitators include partner support and integrated services, while barriers such as limited access and fear of stigma require attention. Youth‐friendly services tailored to young people’s needs improve engagement in care, HIV testing rates, and early linkage to treatment. Adherence support strategies, including counseling, SMS reminders, and peer support clubs, enhance adherence to PrEP and other treatment regimens. Furthermore, this review identified community engagement and empowerment as an effective strategy to strengthen HIV prevention, care, and treatment efforts. IR was crucial in addressing social factors such as poverty, stigma, discrimination, lack of education and awareness, and limited healthcare access. Furthermore, embracing innovative technologies, for instance, mobile health interventions, enhanced service delivery and reach. Critically, these findings highlight that biomedical interventions alone are insufficient to achieve sustained HIV outcomes without addressing underlying social determinants of health. This underscores the need for HIV programs to adopt multisectoral approaches that integrate social, behavioral, and structural interventions alongside clinical services to improve the long‐term impact.

A potential limitation of this review is the reliance on data solely from LMICs in SSA countries, which may restrict the generalizability of the findings to other regions or higher‐income settings. This geographic focus might overlook important variations in healthcare systems, socioeconomic factors, and cultural contexts that could influence the implementation and outcomes of HIV/AIDS prevention, treatment, and care strategies. As a result, the applicability of the review’s conclusions to diverse populations and healthcare settings worldwide may be limited. In addition, the included studies exhibited substantial heterogeneity in study designs, intervention types, outcome measures, and reporting standards, which limited the ability to conduct a formal meta‐analysis and may affect the comparability of findings. The reliance on narrative synthesis, while appropriate, introduces a degree of subjectivity in the interpretation of results. Furthermore, variations in methodological quality and risk of bias across studies may influence the strength and reliability of the conclusions drawn.

Some studies also reported limited implementation outcomes or short follow‐up periods, restricting the assessment of long‐term effectiveness and sustainability of interventions. Contextual differences across settings, including health system capacity, resource availability, and sociocultural factors, may further limit the transferability of findings. Finally, the possibility of publication bias cannot be excluded, as studies with positive findings are more likely to be published. Despite these limitations, this review provides valuable context‐specific insights into implementation strategies for HIV programs in SSA. Future research should prioritize standardized reporting of implementation outcomes, longer follow‐up periods, and more rigorous study designs to strengthen the evidence base and support more robust comparative and quantitative analyses.

## 6. Conclusion

In conclusion, this review of IR studies suggests that DSD and task shifting may be effective approaches for supporting the scale‐up of HIV prevention, treatment, and care. Integrating HIV care with other health services, such as family planning and mental health care, has shown potential for cost‐effectiveness and sustainability, although findings vary across contexts. Strengthening health systems through decentralized HIV testing, including from infancy, may contribute to earlier diagnosis and treatment initiation. Adherence support programs, including counseling and peer support clubs, are associated, in several studies, with improved treatment adherence and retention, although the strength and consistency of evidence differ. Key strategies such as promoting PrEP, implementing test‐and‐treat policies, and preventing mother‐to‐child transmission appear to contribute to HIV prevention efforts, with outcomes influenced by contextual and implementation factors. We recommend continued investment in research to strengthen data monitoring and evaluation, support evidence‐informed decision‐making, identify best practices, and address implementation challenges. Such efforts may help tailor interventions to specific contexts and support the scale‐up of effective strategies for more efficient and impactful HIV prevention and care programs. This may also contribute to strengthening health system capacity through IR.

**Nomenclature**
ART: Antiretroviral therapy
EBI: Evidence‐based intervention
PCR: Polymerase chain reaction
PLHIV: People living with HIV
PMTCT: Prevention of mother‐to‐child transmission of HIV
PrEP: Pre‐exposure prophylaxis
RCT: Randomized controlled trial
WHO: World Health Organization
HCV: Hepatitis C virus
HIV: Human immunodeficiency virus
IR: Implementation research
LMICs: Low‐ and middle‐income countries
MMAT tool: Mixed Methods Appraisal Tool
NIH: National Institute of Health
SSA: Sub‐Saharan Africa.

## Author Contributions

George Msema Bwire participated in study design, protocol development, data extraction and analysis, narrative synthesis, and manuscript drafting. Goodluck G. Nyondo and Belinda J. Njiro participated in protocol development, study screening, data extraction and analysis, risk of bias analysis, and manuscript drafting. Beatrice G. Aiko, Wigilya P. Mikomangwa, Martine A. Manguzu, Francis August, David T. Myemba, and Rejea Magati participated in study design, data analysis, and manuscript revision. Pilly Chillo, Ferdinand Mugusi, Muhammad Bakari, Christopher Sudfeld, Raphael Z. Sangeda, and Japhet Killewo reviewed the narrative synthesis and participated in the manuscript revision.

## Funding

This review received partial support from the Fogarty International Center of the National Institutes of Health (NIH) under Award No.: D43TW009775.

## Disclosure

All authors have read and approved the final version of this manuscript. The funder did not have any role in study design, data collection and analysis, decision to publish, or preparation of the manuscript. The content is solely the responsibility of the authors and does not necessarily represent the official views of the NIH.

## Conflicts of Interest

The authors declare no conflicts of interest.

## Supporting Information

Additional supporting information can be found online in the Supporting Information section.

## Supporting information


**Supporting Information** Supporting file 1. Risk of bias assessment. A large number of studies (22) had a moderate risk of bias, 17 studies had a low risk of bias, and two studies had a high risk of bias (low quality) (Table 1). Many mixed‐methods studies did not adequately address divergencies and inconsistencies between their qualitative and quantitative components as defined by the MMAT tool. Quantitative randomized controlled trials lacked proper blinding of outcome assessors to the intervention. All qualitative studies achieved a high methodological quality (score 5/5), whereas the quantitative nonrandomized and quantitative descriptive studies had a high or moderate risk of bias.

## Data Availability

The data that support the findings of this study are available from the corresponding author upon reasonable request.
